# The FAt Spondyloarthritis Spine Score (FASSS): development and validation of a new scoring method for the evaluation of fat lesions in the spine of patients with axial spondyloarthritis

**DOI:** 10.1186/ar4411

**Published:** 2013-12-16

**Authors:** Susanne Juhl Pedersen, Zheng Zhao, Robert GW Lambert, Stephanie Wichuk, Mikkel Østergaard, Ulrich Weber, Walter P Maksymowych

**Affiliations:** 1 Copenhagen Center for Arthritis Research, Center for Rheumatology and Spine Diseases, Glostrup Hospital, University of Copenhagen, Ndr. Ringvej 57, Glostrup 2600, Denmark; 2 Department of Medicine, Division of Rheumatology, 562 Heritage Medical Research Building, University of Alberta, Edmonton, Alberta T6G 2S2, Canada; 3 Department of Rheumatology, PLA General Hospital, 28# Fuxing Road, Beijing, Haidian District 2600, China; 4 Department of Radiology and Diagnostic Imaging, University of Alberta, 2A2.41 Walter C. Mackenzie Health Sciences Centre, 8440-122 Street Edmonton, Alberta T6G 2B7, Canada

## Abstract

**Introduction:**

Studies have shown that fat lesions follow resolution of inflammation in the spine of patients with axial spondyloarthritis (SpA). Fat lesions at vertebral corners have also been shown to predict development of new syndesmophytes. Therefore, scoring of fat lesions in the spine may constitute both an important measure of treatment efficacy as well as a surrogate marker for new bone formation. The aim of this study was to develop and validate a new scoring method for fat lesions in the spine, the Fat SpA Spine Score (FASSS), which in contrast to the existing scoring method addresses the localization and phenotypic diversity of fat lesions in patients with axial SpA.

**Methods:**

Fat lesions at pre-specified anatomical locations at each vertebral endplate (C2 lower-S1 upper) were assessed dichotomously (present/absent) on spine MRIs. Two readers independently evaluated MRIs obtained at two time points for 58 patients (Exercise 1), followed by optimization of scoring methodology and reader calibration. Thereafter, the same readers read 135 pairs of MRI scans (Exercise 2; including the 58 pairs from exercise 1 randomly mixed with 77 new pairs).

**Results:**

In Exercise 2, the mean (SD) baseline FASSS score for the two readers was 22.5(29.6) and 21.1(28.0), respectively, and the FASSS change score was 4.2(10.6) and 6.0(12.2). Inter-reader reliability assessed as intra-class correlation coefficients (ICCs) for status and change scores were excellent (0.96 (95% CI (0.94 to 0.97)) and very good (0.86 (0.80 to 0.90)), respectively. The smallest detectable change (SDC) was 3.7 for the 135 patients. Good reliability of change scores was also observed for MRI scans conducted one year apart (ICC 0.74 (95% CI 0.44 to 0.89) and SDC 4.5). For the 58 MRI-pairs assessed in both exercises, inter-reader reproducibility for the total FASSS status score improved from very good (ICCs: 0.89 (95% CI: 0.81 to 0.93) in exercise 1 to excellent in exercise 2 (0.96 (0.93 to 0.98)), and improved substantially for the total change score (from 0.67 (0.51 to 0.80) to 0.83 (0.73 to 0.90).

**Conclusions:**

FASSS meets essential validation criteria for quantification of a common structural abnormality in clinical trials of axial spondyloarthritis.

## Introduction

Magnetic resonance imaging (MRI) of the spine in patients with axial spondyloarthritis (SpA) frequently shows focal fat lesions on T1-weighted scans, particularly at vertebral corners and adjacent to the vertebral endplate. Recent studies suggest that fat lesions at vertebral corners may have diagnostic utility in patients with axial SpA [1-3]. Furthermore, focal fat lesions on MRI are more likely to develop at vertebral corners where inflammation has resolved as compared with vertebral corners with persistent or no inflammation at baseline or follow-up [4]. Fat lesions have also been shown to predict development of new syndesmophytes on radiography 2 years later [5]. Consequently, fat metaplasia in the bone marrow of patients with axial SpA may represent an important pathophysiological step in tissue repair after inflammation leading to development of new syndesmophytes and ankylosis. Quantitative assessment of fat lesions on spinal MRI may therefore have utility in the assessment of treatment response as well as constituting a potential surrogate for new bone formation that could be more responsive than radiography.

Of the three scoring methods for structural changes on MRI of the spine in patients with axial SpA reported previously [6-8], only two include assessment of fat lesions [7,8]. Both methods are based on a semi-quantitative assessment of the volume of a disco-vertebral unit affected by fat lesions and do not take into account the anatomical localization and phenotypic diversity of fat lesions. We have therefore developed and validated a new scoring method for focal fat lesions in the spine, the FAt Spondyloarthritis Spine Score (FASSS), which addresses the spectrum of fat lesions according to anatomical localization and phenotypic diversity that can be observed in patients with axial SpA.

## Methods

### Development of the FAt Spondyloarthritis Spine Score

#### *FASSS definitions*

In 2007 a collaboration of Canadian and Danish researchers (the Canada–Denmark MRI working group) developed and validated detailed standardized anatomy-based definitions of inflammatory changes [9,10] and structural changes in the spine of patients with ankylosing spondylitis (AS) [11,12]. These definitions included focal fat lesions at the anterior and posterior vertebral body corners. In 2011 the working group developed further definitions of focal fat lesions according to their anatomical localization at the vertebral endplate when visualized on sagittal MRI slices. The key definitions and characteristics of the lesions assessed in the FASSS are as follows.

First, fat lesion is defined as an increased signal on T1-weighted images. The reference for a normal bone marrow signal is the marrow signal in the center of the vertebral body; if this is not normal, the bone marrow signal of the adjacent most normal vertebra [12].

Second, anterior and posterior vertebral corner fat lesions are located at the vertebral body corners on a central sagittal slice. The latter is defined as a sagittal slice that includes the spinal canal [12].

Third, a noncorner fat lesion is located in a central sagittal slice adjacent to the vertebral endplate but not involving the vertebral corners.

Fourth, a vertebral corner lesion that occurs in lateral slices is named a lateral corner fat lesion. Lateral slices are defined as those slices that do not include the spinal canal and where the pedicle is continuous between the vertebral body and posterior elements or the slice is lateral to the pedicle [12]. The reference structure is the pedicle related to the lower endplate of the disco-vertebral unit (DVU).

Fifth, a corner fat lesion is defined as large if it involves 25% or more of the anterior–posterior diameter of the vertebral endplate and/or the height of the vertebral body. A noncorner fat lesion is defined as large if the lesion involves 25% or more of the height of the vertebral body. If a corner fat lesion in any central slice involves more than 50% of the anterior–posterior diameter of the vertebra, it is considered a combined corner and noncorner fat lesion. Height is measured perpendicular to the endplate.

Finally, all slices at each DVU are assessed systematically for the presence of fat metaplasia. A fat lesion is scored if it is clearly present judged by its size, signal intensity, homogeneity of signal and/or distinct border. The size of the lesion is determined by the size of the lesion on the sagittal slice where it appears largest. For very small lesions, the reader should exercise caution if a subtle observation is only identifiable on one slice.

Examples of fat lesions according to the Canada–Denmark MRI working group are available online [13].

#### *FASSS scoring methodology*

Fat lesions are assessed at each DVU, which constitutes the region between two horizontal lines drawn across the midpoint of adjacent vertebrae in the sagittal orientation. Vertebral corner fat lesions in either central or lateral slices are scored dichotomously (lesion present or absent = 1 or 0). In DVUs in the thoracic and lumbar (but not cervical) spine, a score of 1 is added if a corner fat lesion meets the definition of large (see above) in central slices. Corner and noncorner lesions in the cervical spine are not assessed for size, because the vertebral bodies here are much smaller compared with the vertebral bodies of the thoracic and lumbar segments. By definition, there are no lateral slices in the cervical spine, because the pedicles here are located lateral to the vertebral body and therefore both structures cannot be seen on the same sagittal slices. Corner fat lesions in lateral slices are not assigned a weighting for size, because it is difficult to assess size in relation to anterior–posterior diameter on this curved part of the vertebral body.

**Figure 1:**
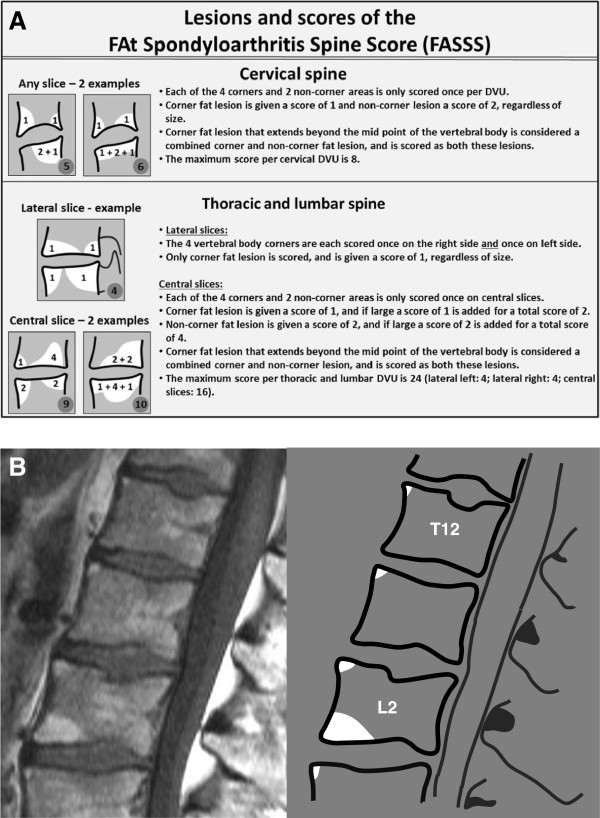
**Different types of fat lesion in the FAt Spondyloarthritis Spine Score. (A)** Types of fat lesions in the FAt Spondyloarthritis Spine Score (FASSS) and the scores applied. Scoring method is described in further detail in the text. **(B)** Corner fat lesions in central sagittal slices. Several focal fat lesions with variable size are located at the anterior corners of the vertebral bodies. The fat lesions at the anterior corner of the upper endplate of T12, L1, L2 and L3 are all small; that is, they do not involve 25% or more of the anterior–posterior diameter of the vertebral endplate and/or of the height of the vertebral body. The fat lesion at the anterior corner of the lower endplate of L2 fulfills the definition of being large (involves ≥25% of the anterior–posterior diameter of the vertebral endplate and/or the height of the vertebral body), and a score of 1 is added. If the fat lesion fulfils the definition of combined corner and noncorner lesion (involves ≥50% of the anterior–posterior diameter of the vertebral endplate), a score of 1 is added if the lesion involves ≥25% of the height of the vertebral body at either the anterior or posterior vertebral cortex. A score of 2 is added if the lesion involves ≥25% of the height of the vertebral body measured at the midpoint of the vertebral endplate. The small corner fat lesions are all scored 1, whereas the large corner lesion is scored 2. The total FASSS for T12–L3 for this single sagittal slice is 6. The score per disco-vertebral unit (DVU) is 1 for T11/T12, T12/L1 and L1/L2, respectively, and is 3 for L2/L3.

Noncorner fat lesions are scored only in central slices and assessed dichotomously (lesion present or absent = 2 or 0). In DVUs in the thoracic and lumbar (but not cervical) spine, a score of 2 is added if the noncorner lesion meets the definition of large (see above). If a corner fat lesion in any central slice involves more than 50% of the anterior–posterior diameter of the vertebra, it is considered a combined corner and noncorner fat lesion. Examples of the different categories of fat lesions and scores are shown in Figure 1. DVUs where the height of the disc is reduced unequivocally by ≥50% are not assessed since the fat lesion here may be caused by coincidental or secondary degenerative disc disease.

Each DVU from C2/C3 to L5/S1 is assessed systematically for the different categories of fat lesions. The scoring range for each thoracic and lumbar DVU is 0 to 24, where the central slices provide a maximum score of 16 (four large corner lesions each scoring 2 = 8, and two large noncorner lesions each scoring 4 = 8) and the lateral slices provide a maximum score of 8 (four right + four left corner lesions each scoring 1). The maximum score for each cervical DVU is 8 (four corner lesions each scoring 1 = 4, and two noncorner lesions each scoring 2 = 4). The scoring range for the total FASSS spine score (all 23 DVUs) is 0 to 456.

### Technical specifications of MRI

MRI was performed at 1.5 Tesla (Siemens, Erlangen, Germany) with appropriate surface coils. Sagittal spine sequences were obtained with 3 to 4 mm slice thickness and 16 to 24 slices were acquired. Sequence parameters were: T1-weighted spin echo (repetition time 423 milliseconds, echo time 13 milliseconds); field of view was 380 mm and matrix was 512 × 256 pixels. Spines were imaged in two parts: an upper part comprising the cervical and most of the thoracic spine, and a lower part comprising the lower portion of the thoracic spine and the lumbar spine. All images were evaluated on dedicated two-monitor (both 25-inch) workstations using DICOM software (ClearCanvas Workstation 2.0 SP1; Canada). The MRIs were anonymized and selected for the study by two technologists not taking part in the study. All MRIs were read independently and in chronological order by two rheumatologists, who were blinded to patient demographics, clinical, and other imaging data.

### Patients and reading exercises

The patients were randomly selected from an observational cohort of consecutive patients with axial SpA (including AS), who had been evaluated systematically according to a standardized protocol including clinical, laboratory, and imaging parameters [14].

Before Exercise 1, both readers were calibrated using the same set of reference images. Data were entered online into a web-based scoring system illustrated as a schematic with upper and lower vertebral endplates for each DVU and diagrammatic representations of the different types of fat lesions. Exercise 1 comprised 58 patients who had two MRIs performed with a mean (standard deviation (SD)) interval of 1.5 (0.5) years. Those scans where readers were most discrepant were discussed by the Canada–Denmark MRI working group. This resulted in further standardization of the definitions and development of reader guidelines for the FASSS.

Two months later, the readers read 135 pairs of MRI scans (Exercise 2) that comprised 58 pairs of scans from Exercise 1 mixed randomly with 77 new pairs of scans with a mean (SD) interval of 1.8 (0.9) years. The purpose of this nested imaging study design was to address the concern that failure to detect improvement may reflect increased difficulty in the case material with different reading exercises. Randomly including case material from the prior exercise and assessing inter-reader reliability for this subset of cases in both exercises provides a more informed estimate of change in reader calibration.

As a further exercise, the two readers 1 year later re-read the 18 pairs of MRI scans (Exercise 3) in which they had been most discrepant in their change scores in Exercise 2. These MRI scans were identified based on pre-specified definitions of discrepancy levels for change scores: an absolute difference in change scores of ≥10, and a relative difference >100% of the mean change score of the two readers (*n* = 8); change scores going in opposite directions (positive vs. negative) with scores ≤ -2 and ≥2 (*n* = 5, two also fulfilled the first definition); and a difference in change scores ≥3 or ≤ -3 if one reader has scored no change (change score 0; *n* = 7).

The study was performed in accordance with the Helsinki Declaration. Written informed consent was obtained from all study participants before inclusion into the observational cohort.

### Statistical analysis

The total FASSS and the segmental FASSS for the two readers were described as mean, SD, median, range, and interquartile range. Inter-observer reproducibility was assessed using intra-class correlation coefficients (ICC). A two-way mixed-effects model with the patient as a random factor and the observer as a fixed factor was used and the results are given as single measures for absolute agreement for baseline and change scores. The smallest detectable change was calculated using the Bland–Altman 80% levels of agreement as recently suggested by Navarro-Compán and colleagues [15]. In contrast to the ICC, which offers an estimate of the relative reliability, the smallest detectable change provides an absolute measure of agreement, which can be used as a guideline for clinicians and applied clinically for assessing real change beyond measurement error at the individual patient level [16].

Reliability analysis was also conducted after stratification according to the time interval between MRI scans (≤1.0 years; >1.0 but ≤1.5 years; >1.5 but ≤2.0 years; >2.0 years). ICC <0.4 was designated fair; ICC ≥0.4 but <0.6 moderate; ICC ≥0.6 but <0.8 good; ICC ≥0.8 but <0.9 very good; and ICC ≥0.9 excellent reproducibility [17]. Reproducibility was assessed with cumulative probability plots and Bland–Altman plots with 80% limits of agreement.

### Ethics

The study was approved by the The Health Research Ethics Board of the University of Alberta, Canada, and was performed in accordance with the Helsinki Declaration. A written informed consent was obtained from all study participants before inclusion into the observational cohort.

## Results

### Patient characteristics

The 58 patients in Exercise 1 did not differ significantly from the 77 additional patients included in Exercise 2 regarding sex (male: 81% vs. 75%), mean (SD) age (40 (13) years vs. 40 (10) years), disease duration (16 (10) years vs. 17 (10) years), Bath AS Disease Activity Index (5.1 (2.0) vs. 5.2 (2.6)), Bath AS Functional Index (4.1 (2.8) vs. 3.9 (2.7)), Bath AS Metrology Index (2.5 (2.1) vs. 2.4 (1.9)) and serum concentration of C-reactive protein (18 (26) mg/l vs. 12 (12) mg/l). Exercise 2 (*n* = 135) comprised 104 (77%) males, with mean (SD) age 40.2 (11.7) years and disease duration 16.9 (10.3) years. Seventy-one (52.6%) of the patients received tumor necrosis factor alpha inhibitors. The 18 patients from Exercise 2 included in Exercise 3 did not differ significantly in baseline characteristics from the patients in Exercise 2 (data not shown).

### Distribution of status and change in the FASSS

Table 1 presents the mean (SD) total and segmental FASSS for Exercises 1 and 2. The highest status (baseline) and change scores were seen in the thoracic spine, followed by the lumbar spine and the cervical spine.

**Table 1:** Total and segmental FAt Spondyloarthritis Spine Scores

	**FAt Spondyloarthritis Spine Score**							
	**Exercise 1 (*n*** **= 58)**				**Exercise 2 (*n*** **= 135)**			
	**Reader 1**		**Reader 2**		**Reader 1**		**Reader 2**	
	**Baseline**	**Change**	**Baseline**	**Change**	**Baseline**	**Change**	**Baseline**	**Change**
Total	15.2 (15.8)	4.5 (12.5)	14.6 (22.2)	4.2 (8.4)	22.5 (29.6)	4.2 (10.6)	21.1 (28.0)	6.0 (12.2)
Cervical	3.5 (4.1)	0.9 (3.4)	1.7 (4.0)	0.2 (0.9)	2.3 (3.9)	0.3 (1.4)	2.1 (4.3)	0.6 (1.7)
Thoracic	8.2 (9.5)	2.7 (8.6)	8.2 (12.6)	2.7 (7.2)	13.4 (19.0)	2.8 (7.8)	11.9 (17.1)	4.0 (9.1)
Lumbar	3.5 (5.3)	0.9 (2.5)	4.8 (8.7)	1.2 (2.8)	6.8 (10.2)	1.1 (3.7)	7.0 (9.7)	1.4 (3.8)

Data are mean (standard deviation) total and segmental FAt spondyloarthritis Spine Scores (as assessed by two readers in Exercise 1 (*n* = 58) and Exercise 2 (*n* = 135).

**Figure 2:**
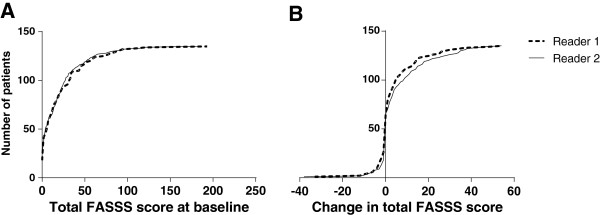
**Probability plots for total FAt Spondyloarthritis Spine Score status and change scores.** Cumulative probability plots for the total FAt Spondyloarthritis Spine Score (FASSS) at baseline **(A)** and for change scores **(B)** for Exercise 2 (*n* = 135).

Figure 2 shows cumulative probability plots for status of (baseline) and change in the FASSS in the Exercise 2 set of scans (*n* = 135 pairs). The median (interquartile range; range) FASSS was 11 (2 to 33; 0 to 193) for Reader 1 and 13 (2 to 28; 0 to 181) for Reader 2. Readers 1 and 2, respectively, found 117 (87%) and 108 (80%) patients with baseline FASSS ≥1. The median change (interquartile range; range) in the FASSS was 1 (0 to 5; -33 to 54) and 1 (0 to 9; -38 to 54). Decreased/unchanged/increased FASSS was observed in 26 (19%)/38 (28%)/71 (53%) patients by Reader 1 and in 18 (13%)/47 (35%)/70 (52%) patients by Reader 2.

### Reliability of status and change in the FASSS

Table 2 presents the inter-observer reproducibility (ICCs) of the total and segmental FASSS in Exercises 1 and 2. In Exercise 1, inter-observer reproducibility of the total FASSS was very good for status and good for change scores. For segmental scores, the inter-observer reproducibility was good to very good for status scores, low to moderate for cervical and lumbar change scores, and very good for thoracic change scores. In Exercise 2, inter-observer reproducibility for both status and change scores was very good to excellent for the total FASSS as well as for segmental scores, except in the cervical spine where the change score improved substantially from low to moderate. The improvements were particularly notable for the 58 patients evaluated in both exercises. The smallest detectable change for the FASSS for the two readers was 3.7 for the 135 patients in Exercise 2. In Exercise 3 where the most discrepant scans were assessed, ICC for status scores remained excellent (0.99 (95% confidence interval: 0.98 to 1.00) vs. 0.91 (0.78 to 0.97)), and the ICC change score improved from 0.04 (95% confidence interval: –0.49 to 0.43) to 0.36 (95% confidence interval: –0.11 to 0.70)).

**Table 2:** Inter-observer reliability of total and segmental FAt Spondyloarthritis Spine Scores

	**Exercise 1 (*n*** **= 58)**		**Exercise 2**					
			**Patients from Exercise 1 re-read (*n*** **= 58)**		**New patients (*n*** **= 77)**		**All patients (*n*** **= 135)**	
	**Baseline**	**Change**	**Baseline**	**Change**	**Baseline**	**Change**	**Baseline**	**Change**
Total	0.89 (0.81 to 0.93)	0.67 (0.51 to 0.80)	0.96 (0.93 to 0.98)	0.83 (0.73 to 0.90)	0.95 (0.93 to 0.97)	0.89 (0.81 to 0.93)	0.96 (0.94 to 0.97)	0.86 (0.80 to 0.90)
Cervical	0.73 (0.39 to 0.87)	0.09 (-0.16 to 0.34)	0.88 (0.81 to 0.93)	0.59 (0.39 to 0.73)	0.82 (0.71 to 0.87)	0.31 (0.09 to 0.49)	0.84 (0.78 to 0.88)	0.37 (0.22 to 0.51)
Thoracic	0.85 (0.76 to 0.91)	0.83 (0.72 to 0.89)	0.93 (0.88 to 0.96)	0.85 (0.76 to 0.91)	0.96 (0.93 to 0.97)	0.86 (0.76 to 0.92)	0.95 (0.93 to 0.96)	0.86 (0.80 to 0.90)
Lumbar	0.76 (0.63 to 0.85)	0.29 (0.03 to 0.51)	0.95 (0.91 to 0.97)	0.75 (0.60 to 0.85)	0.87 (0.81 to 0.91)	0.85 (0.78 to 0.90)	0.91 (0.87 to 0.93)	0.82 (0.76 to 0.87)

Data presented as mean (95% confidence interval). Inter-observer reliability of total and segmental FAt Spondyloarthritis Spine Score as assessed by two readers in Exercise 1 (*n* = 58) and Exercise 2 (*n* = 135), and the 77 new patients included in Exercise 2. The 58 patients from Exercise 1 were nested randomly within the new 77 patients included in Exercise 2.

**Figure 3:**
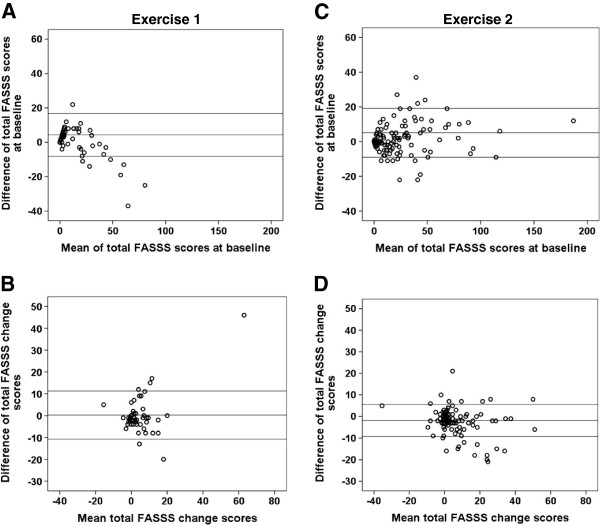
**Bland–Altman plots of FAt Spondyloarthritis Spine Score status and change scores in Exercise 1 and Exercise 2.** Bland–Altman plots of the FAt Spondyloarthritis Spine Score (FASSS) at baseline **(A)**, **(C)** and change scores **(B)**, **(D)** for the two readers in Exercise 1 (*n* = 58) and Exercise 2 (*n* = 135). Horizontal lines represent mean difference and 80% limits of agreement.

**Figure 4:**
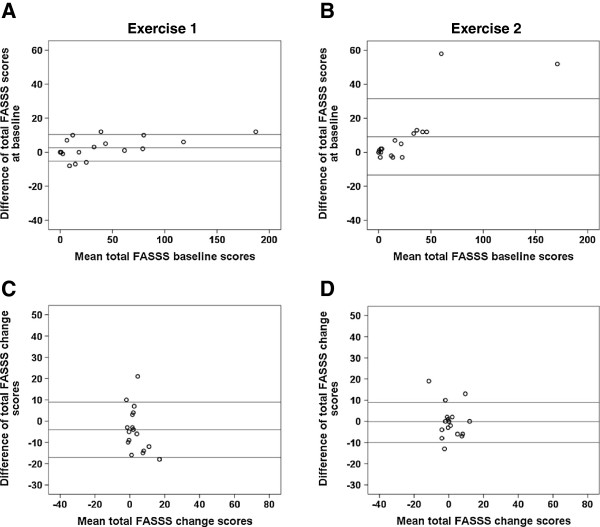
**Bland–Altman plots of FAt Spondyloarthritis Spine Score status and change scores in Exercise 3, first and second reads.** Bland-Altman plots of the FAt Spondyloarthritis Spine Score (FASSS) at baseline **(A)**, **(C)** and change scores **(B)**, **(D)** for the two readers for the 18 most discrepant cases from Exercise 2 according to pre-specified adjudication rules.

Figures 3 and 4 show Bland–Altman plots of the inter-observer differences plotted against the mean of the inter-observer scores. The Bland–Altman plots demonstrated that one reader consistently had higher baseline FASSS in Exercise 1, whereas no systematic differences were seen in Exercise 2 for change scores (Figure 3). Furthermore, the 80% confidence intervals narrowed from the first exercise to the second for the change scores. For the 18 most discrepant patients assessed in Exercise 3, the 80% limits of agreement for the change scores narrowed (Figure 4). For the baseline scores the 80% confidence interval increased due to the results of two outliers, who had spines with a great deal of fat infiltration in the bone marrow and a large number of corner lesions of variable intensity and size. However, the differences between all other scores were very close to 0. Moreover, only five patients fulfilled the definitions for discrepancy after the second read.

### Reliability of the FASSS in relation to time interval between MRI scans

Table 3 presents the baseline and change FASSS for the patients stratified according to the time interval between MRI scans and the corresponding inter-observer reproducibility. Inter-observer reproducibility was very good to excellent, ranging from 0.88 to 0.98, for baseline FASSS. For change in the FASSS, the inter-observer reproducibility was good for the group of patients with MRIs performed within the shortest time interval (≤1 year) and excellent for the two groups with the longest time intervals between MRI scans. The smallest detectable change in the FASSS was between 3.3 and 4.5 for the four time intervals.

**Table 3:** Reliability of FAt Spondyloarthritis Spine Score stratified according to the time interval between MRI scans

**Time interval between MRI scans (years)**		**FASSS**				**Smallest detectable change**	**Reliability**	
		**Reader 1**		**Reader 2**			**ICCs (95% CI)**	
		**Baseline**	**Change**	**Baseline**	**Change**		**Baseline**	**Change**
0 to ≤1.0 (*n* = 20)	0.7 (0.3; 0.3 to 1.0)	21.8 (32.1)	3.7 (6.7)	19.7 (30.2)	6.8 (12.8)	4.5	0.96 (0.89 to 0.98)	0.74 (0.44 to 0.89)
>1.0 to ≤1.5 (*n* = 49)	1.3 (0.2; 1.01 to 1.5)	18.2 (20.9)	4.2 (8.7)	17.3 (19.2)	5.5 (9.5)	3.3	0.88 (0.80 to 0.93)	0.83 (0.72 to 0.90)
>1.5 to ≤2.0 (*n* = 28)	1.9 (0.1; 1.51 to 2.0)	28.8 (40.8)	5.1 (14.0)	27.0 (38.1)	6.7 (15.5)	4.2	0.98 (0.97 to 0.99)	0.90 (0.80 to 0.95)
>2.0 (*n* = 38)	2.6 (0.7; 2.01 to 4.9)	23.9 (28.4)	3.8 (11.9)	22.4 (28.0)	5.8 (12.6)	3.4	0.97 (0.94 to 0.98)	0.90 (0.80 to 0.95)

Time interval presented as mean (standard deviation; range) and FAt Spondyloarthritis Spine Score (FASSS) as mean (standard deviation). CI, confidence interval; ICC, intra-class correlation coefficient; MRI, magnetic resonance imaging. FASSS baseline and change scores for Exercise 2 (*n* = 135 pairs of MRI scans) stratified according to the time interval between MRI scans, agreement and inter-reader reliability for these scores.

## Discussion

In the present study, we describe a new method for scoring fat lesions in the spine of patients with axial SpA and demonstrate that a high degree of reliability can be achieved with minimal calibration for both status and change scores. Most importantly, we show that sufficient reliability can be achieved for change scores even at time intervals of 1 year or less between MRI scans. This indicates that the FASSS deserves further assessment as a prognostic indicator and surrogate for disease progression because reliable detection of the change in the modified Stoke AS Spine Score requires at least 2 years of follow-up [18].

In the FASSS, fat lesions are scored based on multiple anatomical locations in the vertebral body and are scored separately in central and lateral slices with an additional weighting for the size of lesions in central slices. In contrast, two previously reported scoring methods are each based on a semi-quantitative estimate of the fat infiltration for each spinal level that used a 0 to 3 scale to evaluate the size of a lesion according to the anterior–posterior diameter of the area affected. This is performed at 23 spinal levels for a total maximum score of 69. In the Aarhus method [7], fat lesion scores are based on four grades: normal (score 0); slight, <25% of the subchondral bone area of the DVU is affected (score 1); moderate, 25 to <50% is affected (score 2); and severe, ≥50% is affected (score 3). The Berlin method is also based on grading [8], but is applied to the vertebral unit area, which incorporates both sides of a disc. Both the Berlin and Aarhus methods demonstrated good to excellent inter-observer reproducibility for status scores (ICC: FASSS, 0.96 vs. Berlin, 0.97 [8]; Aarhus, κ = 0.68 [7]) when assessed by radiologists, but there are no data available for the reliability of change scores or the smallest detectable change for these systems.

Reliable detection of change scores, particularly within the time frame of clinical trials, is a crucial requirement of any scoring method before implementation in clinical research. Descriptive analyses of FASSS change scores showed that substantial changes occur in patients within a relatively short time interval (mean 0.7 years (that is, 38 weeks)) between MRI scans (3.7 and 6.8 for Reader 1 and Reader 2, respectively). Consistent with our data, the Berlin score increased as early as week 24 in a 48-week randomized trial comparing etanercept with sulfasalazine. The inclusion criteria for this trial required the presence of bone marrow edema on MRI in either the sacroiliac joints or spine [8], whereas no such criteria were used for this study. The same study also demonstrated a significant difference in change scores for the two treatment groups [8]. Knowing that rapid changes in fat score clearly occur, the development of a surrogate outcome that could be discriminatory within a shorter time frame than is required for radiographic discrimination is an exciting prospect. Such a surrogate would be of great benefit as the standard method for reliable detection of change (radiography) requires a 2-year follow-up [18] and it is unethical to maintain randomization for 2 years in trials of disease-modifying therapy [19]. Future studies of prognostic capacity are warranted to determine whether the FASSS could be a valid surrogate for radiographic progression.

Many factors were taken into consideration for the development of the FASSS. The anatomical location and the extent of fat lesions at the perimeter or rim of the vertebra or across the center of the vertebral endplate were regarded as more important for assessment of treatment effects and prognostication than merely quantification of the volume of fat. To improve the measurement of lesions at the vertebral rim, anterior and posterior corner fat lesions located in lateral slices were included in the score. A detailed reliability analyses performed in Exercises 1 and 2 revealed that these lesions were detected with good to excellent reliability for status and change scores, with better reliability when compared with anterior and posterior corner lesions located in central slices (results not shown). The most laterally located fat lesions (that is, noncorner lesions in lateral slices) were less reliably detected on sagittal images and were not included in the scoring system. These latter lesions were also observed very infrequently (<1% of DVU). The lower reliability of detection of lesions in the cervical spine can partly be explained by the different anatomy of the vertebral bodies that extend laterally into the lateral mass without a pedicle, and thereby do not directly fit the definitions. Furthermore, the use of a large field of view, phase-encoding artifacts due to flow phenomena caused by the great vessels in the neck, and the combination of the curvature of the spine and coil artifacts that may cause major variation in signal strength over short distances also all contribute to the lower reliability. Exercise 3 revealed that spines with a great deal of fat infiltration in the bone marrow and a large number of corner fat lesions of variable intensity and size are difficult to score for status (the two outliers in Figure 4B), but not for change. This type of spine is often seen in patients with longstanding disease, where the assessment of fat lesions for prognostication purposes, for example, may be of less value since they already have an ankylosed spine. The inclusion of noncorner fat lesions may seem controversial, since these lesions are often seen in patients with degenerative disc disease (Modic type 2 lesions). However, these lesions are also seen in patients with AS with longstanding disease, where they may be associated with central ankylosis. We therefore consider this type of lesion important for assessment in future studies. However, our scoring system does permit separation of scores for corner lesions from noncorner lesions and thereby allow analysis by lesion type. This flexibility is not available in volume-based methods for assessment of fat lesions.

## Conclusion

We have developed the FASSS, an anatomical-based method to score fat lesions in the spine of patients with axial SpA. We have shown that a high degree of reliability for both status and change scores can be achieved with minimal reader calibration. Most importantly, we show that sufficient reliability can be achieved for change scores even at time intervals of 1 year or less. The FASSS therefore meets essential validation criteria for further assessment in patients with axial SpA in studies of treatment effect and as a surrogate marker for structural damage progression.

## Abbreviations

AS: Ankylosing spondylitis; DVU: Disco-vertebral unit; FASSS: FAt Spondyloarthritis Spine Score; ICC: Intra-class correlation coefficient; MRI: Magnetic resonance imaging; SD: Standard deviation; SpA: Spondyloarthritis.

## Competing interests

The authors declare that they have no competing interests.

## Authors’ contributions

SJP made substantial contributions to the conception and design, read the MRI scans, performed the statistical analyses, interpreted data and prepared the manuscript. ZZ read the MRI scans, participated in the statistical analyses, interpreted data and revised the manuscript. RGWL made substantial contributions to the conception and design, calibrated the MRI readers, and participated in interpretation of data, drafting and revising the manuscript. MO, UW and SW made substantial contributions to the conception and design, interpretation of data, and revision of the manuscript. WPM made substantial contributions to conception and design, participated in data analyses, interpretation of data, preparation and revision of the manuscript. All authors read and approved the final manuscript.
